# Functional Properties and Mechanistic Study of Native Starches as Fat Replacers in Low-Fat Pork Sausages

**DOI:** 10.3390/foods15081428

**Published:** 2026-04-20

**Authors:** Lan Gao, Wentao Chen, Zhenhong Lin, Sitong Ye, Hailin Wang, Guoxin Lin, Daohuang Xu, Chengdeng Chi, Leiwen Xiang, Youcai Zhou

**Affiliations:** 1 School of Food and Biological Engineering, Fujian Polytechnic Normal University, Fuqing, Fuzhou 350300, China; 2 College of Life Science, Fujian Normal University, Fuzhou 350117, China; 3 Xinfengxian Food Co., Ltd., Ningde 352100, China; 4 Fujian Yuguan Food Co., Ltd., Fuzhou 350300, China

**Keywords:** native starches, fat replacers, water-holding capacity, sensory attributes, low-fat meat products

## Abstract

This study systematically evaluated the potential of five native starches, including corn (CS), potato (PS), tapioca (TS), rice (RS), and sweet potato (SPS), as fat replacers in low-fat pork sausages. The obtained results showed that amylose content varied significantly, with PS and SPS having the highest levels (30.06% and 28.60%, respectively), which were beneficial for forming starch gels. Correspondingly, PS and SPS demonstrated the highest solubility and swelling power. In sausage applications, PS and SPS exhibited superior water-retention capacities, with drying losses of 6.75% and 7.03%, and cooking losses of 2.23% and 2.52%, which were lower than those of the normal control (NC) and low-fat control (LFC) groups. Moreover, the results of texture profile analysis revealed that PS and SPS enabled the sausages to achieve the highest levels of hardness and springiness, contributing to maintaining the moisture retention and toughness of the sausages. Electronic tongue and nose analyses indicated that incorporating these starches did not adversely affect the taste and odor profiles of the sausages, except for RS, which showed distinct flavor encapsulation properties. Overall, PS and SPS served as excellent fat replacers in the meat industry, offering healthier alternatives without compromising product quality.

## 1. Introduction

The 2025 Dietary Guidelines for Chinese Residents, aligned with national health policies, promote healthy eating patterns and balanced nutrition. In particular, they recommend restricting the intake of trans fatty acids to improve public nutritional health and reduce the risk of chronic diseases. Pork sausages, a major category of processed meat products, are consumed globally due to their convenience, distinct flavor, and unique textural attributes [1]. Market data indicated that commercial pork sausages contain total fat levels typically ranging from 25% to 40%, including trans fatty acids, cholesterol, and saturated fatty acids. It had been reported that the stable emulsion system formed by animal fats when combined with other ingredients effectively mitigates moisture loss during cooking and baking processes, enhancing water retention and binding capacity [2]. However, excessive consumption of such high-fat products is associated with adverse health outcomes [3]. Epidemiological evidence consistently links the intake of lipid-rich processed meats to an elevated risk of obesity, as well as cancers of the breast, prostate, and colon [4,5,6]. To address these issues, some research has focused on reducing the fat content in conventional sausage formulations, with the goal of decreasing excessive fat intake among consumers [7]. Conversely, reducing fat content can intensify moisture loss during freezing, thawing, and reheating, accompanied by a gradual decline in hardness. This results in a loose structure and deteriorated texture, changes that are generally poorly accepted by consumers [8,9]. Therefore, the development of effective fat substitutes is urgently needed to address these challenges.

In recent years, studies have shown that incorporating various non-meat ingredients, such as protein-based, lipid-based, and starch-based fat substitutes, can effectively reduce animal fat content in processed products without compromising their structural integrity, flavor, or sensory quality, while posing no known adverse effects on human health [10,11,12]. However, protein-based fat substitutes have poor thermal stability. When incorporated into meat sausages and subjected to thermal processes such as steaming, baking, or grilling, they often undergo undesirable alterations in flavor and texture, thereby limiting their practical application [13]. Lipid-based substitutes, on the other hand, can reduce caloric content without substantially compromising the sensory properties or appearance of food products [14]. Nevertheless, certain lipid-based alternatives resist hydrolysis by digestive lipases, which prevents their metabolism and absorption and may consequently raise gastrointestinal concerns [15]. In comparison, starch-based fat substitutes demonstrate superior safety under high-temperature food processing conditions relative to protein- and lipid-based alternatives [16]. Due to their cost-effective sourcing and favorable functional properties, starch-derived fat substitutes have attracted considerable research interest. Incorporating one or more low-calorie, starch-based alternatives into sausage formulations can therefore support the development of reduced-fat meat products that align with growing consumer demand for healthier options [17].

Starch, a polymer consisting of α-D-glucose units, encompasses both native and modified forms, and it possesses distinct physicochemical properties, such as thickening ability, viscous flow behavior, puffing capacity, water retention, and gel formation [18,19]. Certain starch granules exhibit size and shape similarities to fat globules, enabling their dispersion within food systems to deliver sensory attributes comparable to those imparted by fat [20]. In recent years, research has focused extensively on the modification of native starch [21,22]. By using physical, chemical, or enzymatic treatments, functional groups can be introduced onto starch molecules, or granule size and properties altered, thereby enhancing the functional characteristics of starch for use as a fat substitute [23]. However, these modification processes often involve complex procedures, extended processing times, and increased production costs [24]. In contrast, native starch, in its unmodified form, offers a more natural and safer alternative as a fat substitute and may reduce overall production costs, making it particularly suitable for large-scale application in pork sausage formulations [25,26]. To the best of our knowledge, there is limited systematic research comparing the specific application effects of readily available native starches in meat products or elucidating the underlying mechanisms by which the hydrophilic properties of these starches influence sausage quality when used as fat substitutes.

With this background, the aims of this study were to (1) systematically evaluate five native starches (corn starch (CS), potato starch (PS), tapioca starch (TS), rice starch (RS) and sweet potato starch (SPS)) as fat substitutes in low-fat pork sausages; (2) analyze their structural properties and correlate them with key quality indicators, including water holding capacity, cooking loss, drying loss, and sensory attributes; (3) sensory evaluation of five types of starch applied in pork sausages by electronic tongue and nose. The findings will provide essential and vital scientific details to develop low-fat meat products.

## 2. Materials and Methods

### 2.1. Materials

Corn starch (CS), potato starch (PS), tapioca starch (TS) and sweet potato starch (SPS) were purchased from SuZhou GaoFeng Starch Technology Co., Ltd., Suzhou, China. Rice starch (RS) was purchased from Zhejiang Yicun Biotechnology Co., Ltd., Hangzhou, China. Fresh pork hind leg meat and pork back fat were purchased from the local supermarket (Yonghui Supermarket, Fuqing, Fuzhou, China). Before use, the pork back fat and the removed surface fat and fascia from the pork hind legs were cut into small pieces, then ground with a food processor, and stored at −20 °C.

### 2.2. The Preparation of Starch and Pork Emulsions

The frozen pork hind leg meat was thawed in a 4 °C refrigerator for 12 h. As a pretreatment, 500 g of the pork hind leg meat was mixed with 100 g of ice water (4 °C), and then chopped at 1500 rpm for 30 s. The experiments to determine the water and oil retention of the meat were conducted with different starch groups (CS, PS, TS, SPS and RS), a normal control group (NC), and a low-fat control group (LFC) (see Appendix A, Table A1).

For the different starch groups, 60 g of the treated pork hind leg meat, 21 g of pork backfat, 10 g of ice water, and 9 g of each different starch sample were used. The normal control group (NC) consisted of 60 g of treated pork hind leg meat, 30 g of pork backfat, and 10 g of ice water. The low-fat control group (LFC) contained 69 g of treated pork hind leg meat, 21 g of pork backfat, and 10 g of ice water. Each of these treatments was chopped for 30 s, followed by a resting period of 3 min, and then finally chopped again for an additional minute. The temperature during the chopping periods did not exceed 10 °C.

The ground starch–meat mixture was stuffed into the casings, and a fine needle was used to prick small holes in the sausage surface to prevent rupture from excessive internal pressure during cooking. Subsequently, the sausages were boiled in 100 °C water for 15 min. After cooling to room temperature, the drying loss, cooking loss, color difference, hardness, and sensory characteristics of the sausages were measured.

### 2.3. Determination of the Pasting Properties of Starch

The pasting characteristics of starches were evaluated by a Rapid Visco Analyzer (RVA-4500 by Perten Instruments), as previous study [27].

### 2.4. Analysis of Starch Structure

The crystalline structure of starch samples was tested using an X-ray multi-crystal powder diffractometer (XRD) (Rigaku/MiniFlex II, Rigaku Corporation, Tokyo, Japan), as in our previous report [28].

The chain length distribution of the five starches has been determined as described in our previous report [29]. The chromatograph used in this study was supported by Sanshu Biotech. Co., Ltd. (Shanghai, China). Briefly, the starch sample (5 mg) was thoroughly mixed with 5 mL of DMSO solution containing 0.5% LiBr (*w*/*w*) and then heated at 90 °C using a thermomixer for 3 h. The molecular weights of different starch samples were determined using gel permeation chromatography (GPC) technology with a refractive index detection (SEC-RI). The GPC system employed a gel permeation chromatography-differential refractometer system. The liquid chromatography system was a U3000 (Thermo, Waltham, Massachusetts, CA, USA), and the differential refractive index detector was an OPTILAB T-rex (Wyatt Technology, Santa Barbara, CA, USA). Based on the properties of starch, gel exclusion chromatography columns with appropriate molecular weight ranges were used for testing: Plael 10 μm MIXED-B (300 × 7.5 mm) and Plgel 5 μm MIXED-D (300 × 7.5 mm) (Agilent Technologies, Santa Clara, CA, USA). Dextran standards with established molecular weights (342, 3650, 21,000, 131,400, 610,500, 821,700, and 3,755,000 Da) were used for column calibration. The column temperature was set at 80 °C; the injection volume was 100 μL; the mobile phase was DMSO containing 0.5% (*w*/*w*) LiBr, with a flow rate of 0.8 mL/min, and the elution gradient was isocratic for 60 min.

### 2.5. The Determination of Solubility and Swelling Power of Starches

We accurately weighed a starch sample and recorded its mass (M_0_). We dispersed the starch sample in 50 mL of distilled water to prepare a homogeneous suspension, then placed the suspension in a constant-temperature water bath, heated it to 95 °C, and maintained this temperature for 30 min, continuously stirring during this period to ensure uniform heating. After heating, the suspension was transferred into centrifuge tubes and centrifuged at 3000 rpm for 15 min to allow undissolved starch particles to sediment for 30 min. We carefully aspirated the supernatant and transferred it to a pre-dried and pre-weighed evaporating dish. The evaporating dish was placed in a 105 °C oven and dried until a constant weight was achieved, then we recorded the mass of the residue (M_1_).

(1) $$Solubility\;(\%)=\frac{M_{1}}{M_{0}}\times 100\%$$

We weighed approximately 1 g of starch sample and recorded its mass as M_2_. We placed the sample into a pre-dried and pre-weighed centrifuge tube. We then added 20 mL of distilled water to the centrifuge tube to prepare a starch suspension, placed the centrifuge tube in a 25 °C constant-temperature water bath, and kept it for 30 min to allow the starch to fully absorb water and swell. Then, the centrifuge tube was centrifuged at 3000 rpm for 15 min to sediment the unswollen starch particles. We carefully poured out the supernatant and used filter paper to blot dry any residual liquid at the mouth of the centrifuge tube. The total mass of the centrifuge tube and the swollen starch (M_3_) was weighed. The swelling power (g/g) was calculated using the following equation:

(2) $$Swelling\;(g/g)=\frac{M_{3}-M_{2}}{M_{2}}$$

### 2.6. Water-Holding Capacity of Starch-Based Sausages

The water holding capacity of starch-based sausages was determined by the two indices (drying loss and cooking loss) following the report by Cheetangdee N [30]. Briefly, sausage slices with a uniform thickness of 5 mm were dried in an oven at 60 °C for 20 min. The mass of each sample was measured before and after drying and recorded as M_1_ and M_2_, respectively.

(3) $$Drying\;loss\;(\%)=\frac{M_{1}-M_{2}}{M_{1}}\times 100\text{\%}$$

The sausages were wiped with filter paper to remove surface moisture and fat, then weighed, with the mass recorded as M3. The sausages were then placed in a water bath at a constant temperature of 80 °C and heated for 30 min. After removal from the water bath, the sausages were cooled to room temperature, the surface moisture and fat were wiped off with filter paper, and the sausages were weighed again, denoted as M4.

(4) $$Cooking\;loss\;(\%)=\frac{M_{3}-M_{4}}{M_{3}}\times 100\text{\%}$$

### 2.7. Determination of Texture and Odor Characteristics of the Sausage

The texture characteristics of the sausage were determined by an electronic tongue (SA402B, Intensor Teelligent Technology, Atsugi, Japan) followed previous study [31]. Briefly, the sausage samples (10 g) were placed in a beaker, 100 mL of distilled water was added, and the mixture was stirred well. Then, the samples were subjected to ultrasonic treatment (200 w, 25 °C) for 30 min for gas enrichment. The mixture was filtered through filter paper to collect the filtrate for subsequent testing. The analysis conditions for the electronic tongue were: data acquisition for 120 s, followed by a cleaning cycle of 180 s.

The odor characteristics of the sausage were tested using an electronic nose (PEN3, AirSense Technology) [32]. The sausage samples (3 g) were placed in a measuring cup. The cup was then sealed and allowed to stand for 30 min for gas enrichment. The analysis conditions for the electronic nose (PEN3, AirSense Technology, Schwerin, Germany) were as follows: a flow rate of 400 mL/min was delivered to the detection chamber, with a sampling duration of 120 s.

### 2.8. Determination of Color Difference in Starch-Based Sausages

The color difference in starch-based sausages was measured using a fully automatic color difference meter (Ci4200 Color Difference Detector, X-Rite, Grand Rapids, MI, USA), determining the lightness (L* value), red-green range (a* value), and blue-yellow range (b* value). The sausage samples were sliced into cylindrical sections with a height of 20 mm. Prior to measurement, the instrument underwent self-checks and was calibrated using both zero-point and standard white plate adjustments. After removing the casings, the sausages were minced into a homogeneous meat paste. This paste was then evenly distributed across the bottom of the sample cell to ensure uniform coverage before proceeding with the measurements.

### 2.9. Determination of Hardness of Sausages

The hardness of sausage samples was measured using a texture analyzer (EZ-SX, Shimadzu Corporation, Kyoto, Japan). The sausage samples were sliced into cylindrical shapes with a diameter of 20 mm and a height of 10 mm, ensuring uniformity in sample shape and dimensions to minimize the impact of shape variations on the measurement results. Compression testing was conducted to record the compressive force values and the degree of sample deformation, followed by calculation of hardness. A P/50 probe with its corresponding platform was used, with the following specific test parameters: pre-test speed of 5.0 mm/s, test speed of 2.0 mm/s, post-test speed of 2.0 mm/s, a time interval of 5 s, and a compression level of 50%.

### 2.10. Statistical Analysis

All data were expressed as the mean ± standard deviation (SD), and analyzed using SPSS version 23.0 (SPSS Inc., Chicago, IL, USA). Origin 8.5 software was selected to analyze the data. One-way analysis of variance (ANOVA) was performed and followed by Dunnett’s multiple comparison test. The *p*-value < 0.05 was considered statistically significant.

## 3. Results and Discussion

### 3.1. Relative Molecular Weight and Chain Length Distributions of Five Starches

**Figure 1:**
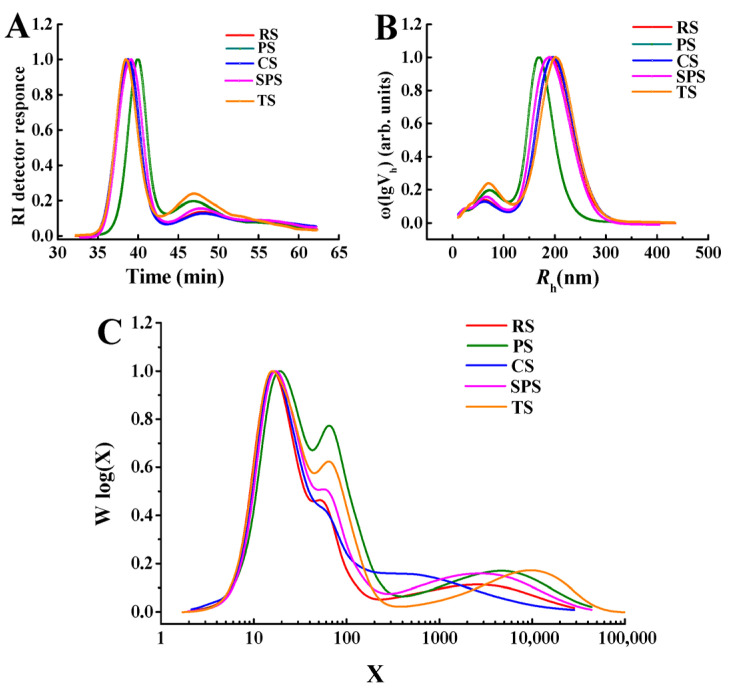
Differential refractive index response curves of the five starches obtained via gel permeation chromatography (**A**), the chain-length distributions of debranched starches (**B**), and logarithmic distribution of weight fractions (**C**). Note: RS, PS, CS, SPS, TS represent rice starch, potato starch, corn starch, sweet potato starch, and tapioca starch, respectively.

As shown in Figure 1, the molecular weight distribution of five starches and their corresponding chain-length distributions of amylose and amylopectin were analyzed using SEC-RI. There were significant differences in the peak signal intensities of different samples within the time ranges of 35–43 min and 43–55 min, indicating notable variations in molecular weights among the samples (Figure 1A). Figure 1B displays the hydrodynamic radius (*R_h_*) distributions of the five starch samples (RS, PS, CS, SPS, TS). The *R_h_* represented the apparent size of starch molecules in solution, reflecting their molecular dimensions and conformational characteristics. All five starches exhibited a major peak in the *R_h_* range of 100–400 nm, suggesting similar but distinct dominant molecular sizes (Figure 1B). The differences in peak positions and shapes among the starches reflected variations in their molecular weight distributions and aggregation behaviors. For instance, TS showed a sharper peak compared to RS (cereal starch), implying a more uniform molecular size distribution in tuber starches (Figure 1B). These variations in distributions are closely related to the functional properties of starches, such as thickening, gelling, and adhesive behaviors in food applications [33].

Figure 1C illustrates the chain length distributions of the five starch samples, represented as *Wlog*(*X*), where *X* was the degree of polymerization (DP, i.e., chain length). Obviously, the heterogeneity in the molecular chain lengths within each starch type was significant. All starches exhibited a broad distribution, with a major peak around *log*(*X*) = 1 (e.g., *X* = 10), suggesting a high abundance of shorter chains. Specifically, the higher prevalence of longer chains in tuber starches (e.g., TS and PS) contributes to their superior thickening and adhesive properties, making them more suitable for industrial applications. For instance, in meat products, these characteristics can influence the water retention, texture, and stability of the products [34]. Commonly, cereal starches contain a greater proportion of low molecular weight fractions compared to tuber starches, whereas tuber starches are characterized by a higher prevalence of high molecular weight fractions. As a result, tuber starches (TS and PS) are the favored option for the production of food thickeners or adhesives.

Table 1 presents the chain-length distribution of five starch samples. It was clear that the amylose content exhibits variability among the five samples. PS had the highest amylose content at 30.06%, while RS had the lowest among them at 21.93%. As for the chain-length ranges, in the short-chain segment (X ≤ 13%), PS displayed a notably lower proportion (10.73%) compared to the other starches. This suggested that PS has a relatively reduced abundance of very short chains, which could potentially affect its initial hydration and swelling behavior during processing [35]. Furthermore, in the range of 13 < X ≤ 24, all starches showed relatively elevated percentages. However, RS had a relatively high value of 27.89%, indicating that RS has a more pronounced presence of chains within this specific length interval. This may contribute to its unique pasting and textural characteristics, which are consistent with the phenomena stated by previous works [36]. Moreover, PS attained 25.80% of longer chain-length intervals (36 < X ≤ 100), signifying a greater proportion of medium–long chains. These medium–long chains are often associated with the formation of more stable gel networks and can influence the firmness and elasticity of starch-based products [37]. It had been reported that the gel network formed by the medium–long chains of starch can encapsulate the meat paste, enhancing the water-holding capacity and elasticity of the meat products and making their texture more resilient [38]. The gel structure is also more stable, making the products less prone to quality issues such as water separation and softening during storage and transportation. Therefore, PS served as an excellent material for fat substitutes in sausages.

### 3.2. Crystalline Structures of the RS, TS, PS, SPS, and CS

**Figure 2:**
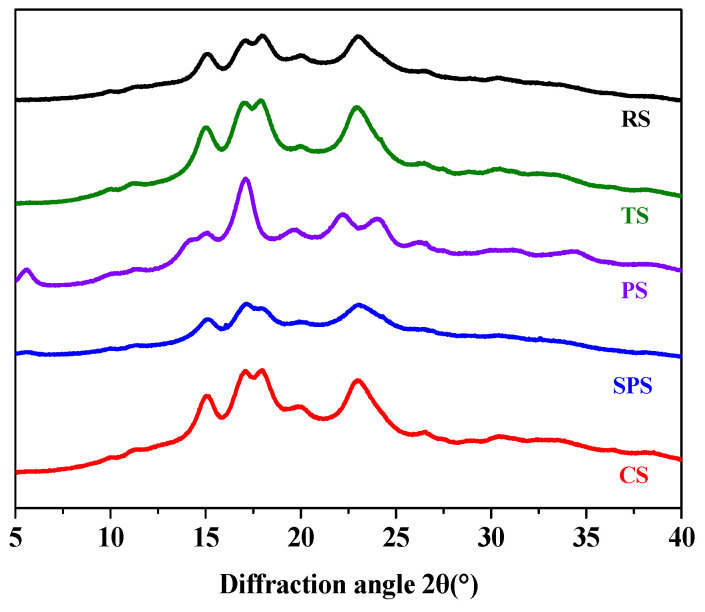
X-ray diffraction patterns of five starches. Note: RS, PS, CS, SPS, TS represent rice starch, potato starch, corn starch, sweet potato starch, and tapioca starch, respectively.

X-ray diffractometers were used to study the crystal structures of RS, TS, PS, SPS, and CS, and the results are shown in Figure 2. In the analysis of the XRD pattern, RS, TS, and CS exhibited distinct single peaks at 15° and 23° 2*θ*, with connected double peaks at 17° and 18° 2*θ*. These characteristics indicated that the three types of starch belong to A-type starch, which has a relatively compact crystal structure with high order and small internal pores [34]. This structure allows them to bind less water but exhibit excellent water-holding capacity. On the other hand, the PS sample showed a prominent peak at 2*θ* = 5.6°, absent in other starches, and weak diffraction peaks at 2*θ* = 15°, 20°, and 24°, which had the lowest crystallinity (14.5%) compared to other starches (see Appendix A Table A2). Therefore, PS was classified as B-type starch, characterized by a more loosely packed crystalline structure and lower structural order compared to A-type crystals. This arrangement allowed PS (B-type starch) to have a loose crystalline structure, which contributed to its strong water binding capacity [39]. Meantime, SPS exhibited a characteristic peak at 5.6° 2*θ*, connected double peaks at 17° and 18° 2*θ*, and simultaneously possessed diffraction peaks characteristic of both A-type and B-type starches (Figure 2). This indicated that SPS belongs to C-type starch, whose crystal structure is intermediate between A-type and B-type, combining the performance characteristics of both types of starch.

### 3.3. Gelatinization and Functional Properties of the Five Starches

**Figure 3:**
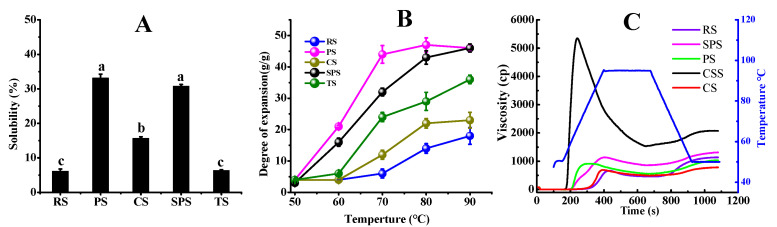
Solubility (**A**), swelling power (**B**) and pasting viscosity profiles (**C**) of five starches. Note: values with different letters are significantly different (*p* < 0.05). Note: RS, PS, CS, SPS, TS represent rice starch, potato starch, corn starch, sweet potato starch, and tapioca starch, respectively.

Starch gelatinization refers to the process in which starch granules absorb water, expand in volume, and ultimately form a viscous colloidal solution during heating [40]. Due to differences in molecular structure and the ratio of amylose to amylopectin, starches from different sources exhibit varying gelatinization characteristics. During starch gelatinization, the interaction between starch molecules and water molecules changes, thereby affecting the hydrophilicity of the starch. Figure 3 presented the solubility and swelling power of starches extracted from different plant sources. Among the evaluated starches, PS exhibited the highest solubility (33.17%), which can be attributed to its loose B-type crystalline structure, both of which enhance water dispersibility, which aligned with previous reports [41,42]. In contrast, RS showed the lowest solubility (6.17%), due to its higher amylose ratio that restricts granular hydration (Figure 3A). Regarding swelling power, PS demonstrated the most pronounced swelling capacity, 46.11 g/g, resulting from its larger granule size, high phosphate content, and weak associative forces within the amorphous regions (Figure 3B).

As shown in Figure 3C, RS exhibited a higher initial gelatinization temperature, subsequently rising to nearly 1000 cp, and after a temperature drop, its viscosity increases slightly. On the other hand, TS demonstrated a lower initial gelatinization temperature, with its viscosity rapidly increasing to around 5000 cp as the temperature changes, then gradually decreasing to approximately 2000 cp and remaining relatively stable thereafter. Table 2 showed the paste properties of the five starches. Notably, the paste temperatures of RS, PS, CS, SPS, and TS are 90.55, 76.00, 92.65, 75.30, and 68.55 °C, respectively. Meantime, PS and SPS showed the setback viscosity values of 478.56 and 455.19 mPa⋅s, which were significantly lower than those of RS and TS, but higher than CS (Table 2). Hou C et al. [43] demonstrated that treating commercial wheat starch (98.85% starch content, 23.4% amylose) with low molecular weight arabinoxylan (L-WEAX) and maintaining a solubility of 14.03% and swelling power of 13.11 g/g suppressed amylose leaching and amylose-lipid complex formation, thereby exhibiting a more pronounced inhibitory effect on starch gelatinization and short-term retrogradation. In the presented study, PS and SPS exhibit higher solubility, swelling power, viscosity stability and lower setback viscosity during heating, which are associated with their higher amylose content and hydrophilic granular structure. These characteristics contribute to moisture retention during thermal processing such as cooking, highlighting the decisive influence of botanical origin and molecular composition on starch functionality.

### 3.4. The Effects of RS, TS, PS, SPS, and CS on the Water Loss of Sausages

**Figure 4:**
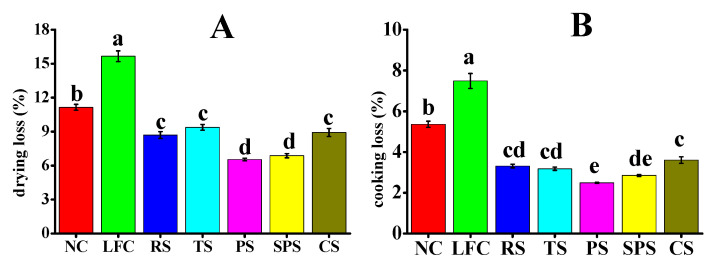
Drying loss (**A**) and cooking loss (**B**) of sausages formulated with the five starches. Note: Values with different letters are significantly different (*p* < 0.05). NC, LFC, RS, PS, CS, SPS, TS represent normal control, low-fat control, rice starch, potato starch, corn starch, sweet potato starch, and tapioca starch treatments for pork sausages, respectively.

The water-holding capacities of the five starches for pork sausages were tested using the indices of drying loss and cooking loss, and the results are shown in Figure 4. Obviously, all starch varieties significantly reduced moisture loss in sausages compared to the no-starch control (NC) group, demonstrating consistent trends in both drying loss and cooking loss (Figure 4). The PS and SPS exhibited the best water-holding capacities, with drying loss values of 6.75% and 7.03%, and cooking loss values of 2.23% and 2.52%, respectively. The cooking and drying loss rates were ranked as follows: LFC > NC > TS > CS > RS > SPS > PS. Moisture loss is a crucial factor that results in the drying and disintegration of sausage structure, ultimately impacting the food’s flavor and overall quality [44]. Gómez-Estaca et al. [45] used the oil-based fat replacers gelled with beeswax and ethyl cellulose in pork burgers and showed cooking losses of 18.2–20.5% and drying losses of 6.8–8.3% that were lower than those of full-fat controls. In the study of Zampouni K et al. [46], the olive oil oleogel was used as a fat replacer in fermented sausages, which resulted in cooking losses of 12.1–14.3% and drying losses of 7.5–9.2%. In this present study, the drying loss and cooking loss of PS and SPS were lower than those reported by Gómez-Estaca et al. and Zampouni et al. Notably, PS and SPS exhibited lower moisture exudation rates in practical applications due to their exceptional hydrophilicity, highlighting their ability to effectively retain moisture during baking and cooking processes. This, in turn, contributed to the preservation of moisture content and structural integrity in sausages. The ability of starches to reduce moisture loss can be attributed to their interaction with water molecules and the protein matrix within the sausages [47]. Starches with high water-binding capacities, such as PS and SPS, likely form more stable gels or complexes (Figure 3C) with water during the heating and drying processes, which is in line with their previous results of crystal structures and pasting properties.

On the basis of the results in Figure 4, PS and SPS could be developed into an excellent starch-based fat substitute for sausages. However, it is worth noting that the choice of starch should not only be based on moisture retention ability but also on other factors such as the texture and odor characteristics of the sausage. Therefore, the following experiments, which involved the use of an electronic tongue and an electronic nose, were conducted to evaluate the texture and odor characteristics of the starch-based sausage.

### 3.5. The Flavor Characteristics of Starch-Based Sausages

#### 3.5.1. Electronic Tongue Test of Sausage

**Figure 5:**
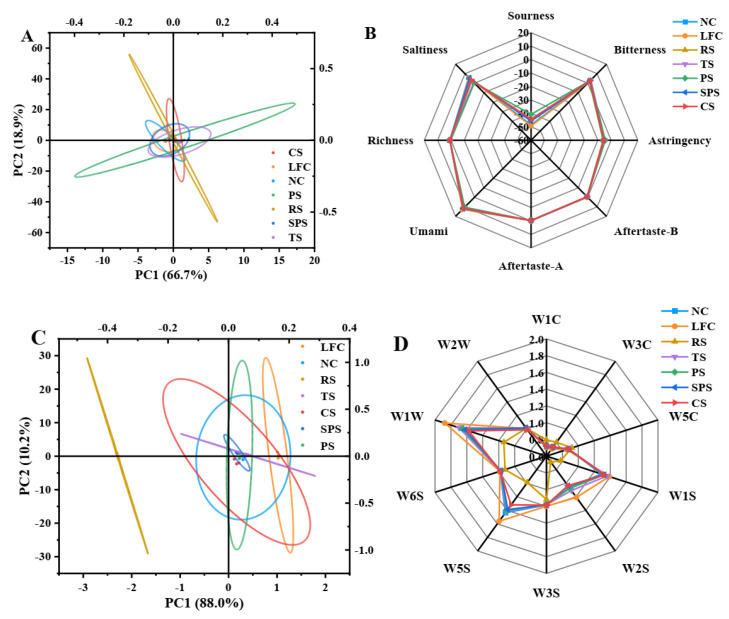
Taste properties of the sausages: score plot (**A**) and radar chart (**B**) determined by the electronic tongue; score plot (**C**) and radar chart (**D**) determined by the electronic nose. Note: NC, LFC, RS, PS, CS, SPS, TS represent normal control, low-fat control, rice starch, potato starch, corn starch, sweet potato starch, and tapioca starch treatments for pork sausages, respectively.

Figure 5A presents the principal component analysis (PCA) of the electronic tongue data for various starch meat sausage samples. The results indicated that the contribution rate of the first principal component (PC1) was 66.7%, and that of the second principal component (PC2) was 18.9%, suggesting that the data after dimensionality reduction by principal components can reflect the overall information of the original data. It can be observed that the regions where each sample was located overlap and cluster (Figure 5A), indicating that their taste characteristics share certain similarities.

In the analysis of specific sensor response values, it was observed that the sourness sensor in the LFC group exhibited significantly higher response values (Figure 5B). This phenomenon may be associated with insufficient emulsification stability in the sausages from the LFC group. Specifically, during sausage processing, inadequate emulsification stability could lead to the separation of moisture and fat, thereby increasing the risk of acidification in the sausages. This process may serve as a critical factor contributing to the elevated response values observed in the sourness sensor. The present study revealed that insufficient emulsification stability in LFC group sausages was likely a key determinant of their abnormal sourness characteristics. Differently, the problem of insufficient emulsification stability can be effectively addressed in sausages formulated with five types of starch. This finding carried important implications for optimizing sausage processing techniques and enhancing product quality.

#### 3.5.2. The Electronic Nose Test of Sausage

Figure 5C presents the results of PCA performed on electronic nose data from various starch-based sausage samples. As shown in Figure 5C, PC1 accounted for 88.0% of the variance, while PC2 explained 10.2%, indicating that the dimensionality-reduced data effectively retained the overall information of the original dataset. Meantime, the RS group showed significant separation from other groups, while the sample regions of the remaining groups were closely clustered. This suggested that apart from the RS group, other groups exhibited high similarity in olfactory characteristics. According to a previous study, encapsulation structures using RS as the matrix can effectively retain flavor compounds [31]. Thus, we hypothesized that this encapsulation mechanism might be the primary reason for the distinct flavor profile observed in rice starch-based sausages compared to other groups.

During the analysis of response values from specific sensors (Table 3), it was observed that the W1W, W1S, and W5S sensors in the LFC group demonstrated significantly higher response values (Figure 5D). These discrepancies were primarily manifested in the detection of sulfur compounds, alcohols, and nitrogen oxides, which may correlate with insufficient emulsion stability in the LFC group. Specifically, during processing, inadequate emulsion stability led to substantial lipid leakage, followed by oxidative reactions, which could serve as critical factors contributing to the abnormally high sensor response values.

These findings indicated that starch types, with the exception of RS, have minimal impact on the flavor profiles of meat sausages and do not lead to significant differences in flavor evaluations. However, the insufficient emulsion stability in the LFC group further compromises its olfactory characteristics.

### 3.6. Effects of Different Starch Additions on Sausage Color Difference

Although the color of meat products does not affect their nutritional value, this characteristic directly affects consumers’ sensory perception and consequently their product acceptance. Obviously, the addition of starch significantly enhanced the luminance value of sausages (Table 4). Starch molecules reflect specific light wavelengths due to their structure. Adding starch to sausages changes their optical properties, enhancing light reflection and increasing brightness [25]. Among them, adding PS and SPS raised the brightness value of sausages quite noticeably, with the values reaching 59.16 and 58.79, respectively. This phenomenon could be explained by the fact that, during gelatinization, PS and SPS have excellent gel properties (Figure 4). These properties enable them to fill gaps in sausages, making the surfaces smoother. The smooth surfaces then reflect light regularly, reducing scattering and absorption, so the sausages look brighter.

In terms of redness values, the addition of five types of starch reduced the redness of sausages to varying degrees. However, the LFC group exhibited the lowest redness value among all experimental groups. The possible reason is that during the cooking process, sausages with fat directly removed experience significant water loss. Since water absorbs red light more strongly than blue light, this leads to a reduction in the redness value of the sausages. Regarding the yellowness index, the addition of starch reduced the yellowness value of sausages, whereas in the LFC group, the yellowness value of sausages actually increased.

### 3.7. The Texture Properties of Sausages Influenced by Starches

**Figure 6:**
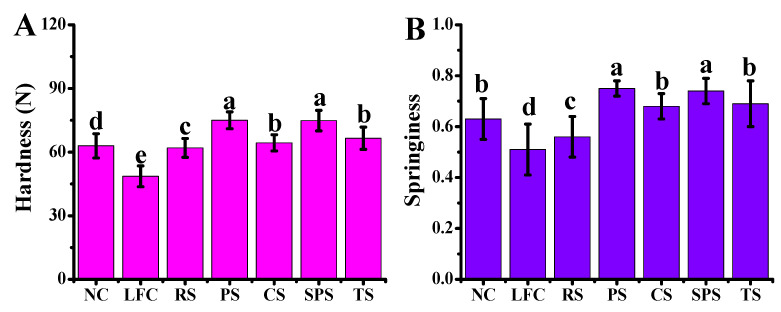
The textural characteristics in sausages derived with varying starches ((**A**) Hardness; (**B**) springiness). Note: values with different letters are significantly different (*p* < 0.05). NC, LFC, RS, PS, CS, SPS, TS represent normal control, low-fat control, rice starch, potato starch, corn starch, sweet potato starch, and tapioca starch treatments for pork sausages, respectively.

Texture characteristics are crucial indicators for evaluating the mouthfeel and texture of sausages, typically assessed through parameters, such as hardness and springiness. In this study, compared to the NC and LFC groups, five types of starch increased the hardness of the sausages to some degree (Figure 6A). Among them, the addition of PS and SPS resulted in the highest hardness values of 74.97 N and 74.83 N, respectively. This notable increase in hardness can be attributed to the unique molecular structures and gel-forming abilities of PS and SPS. The high amylose content in these starches contributes to the formation of a more stable and rigid gel network during the cooking process, thereby enhancing the structural integrity and firmness of the sausages. On the other hand, the PS and SPS also significantly enhanced the springiness of the sausages, with the values reaching 0.75 and 0.74, respectively (Figure 6B). Springiness, which reflects the ability of the sausage to recover its original shape after deformation, is a critical parameter for consumer acceptance, especially in products that are expected to have a juicy and tender texture [48]. Commonly, highly elastic sausages can generally better retain meat juice and prevent excessive water loss during cooking [49]. The improved springiness observed in sausages formulated with PS and SPS can be linked to the excellent water-retention capacity of these starches. By effectively retaining moisture within the sausage matrix, PS and SPS helped to prevent the sausage from becoming overly dry and tough, thus maintaining its elasticity and resilience. Furthermore, the ability of PS and SPS to enhance both hardness and springiness simultaneously was particularly advantageous in the context of low-fat sausage production. Reducing fat content in sausages often leads to a decline in texture quality, as fat plays a crucial role in providing juiciness, tenderness, and mouthfeel. However, the incorporation of PS and SPS as fat replacers can effectively mitigate these negative effects by forming stable gel networks that mimic the functional properties of fat. This not only improved the overall texture of the sausages but also aligned with the growing consumer demand for healthier meat products with reduced fat content.

### 3.8. Interrelationship Between the Determined Starch Characteristics and Water-Holding Capacities of Pork Sausage

**Figure 7:**
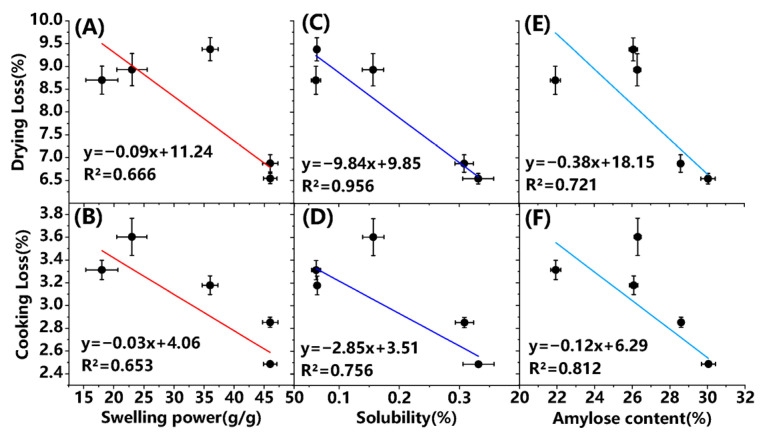
The interrelationship between starch characteristic and water-holding capacity using linear equation. (**A**), swelling power with drying loss; (**B**), swelling power with cooking loss; (**C**), solubility with drying loss; (**D**), solubility with cooking loss; (**E**), amylose content with drying loss; (**F**), amylose content with cooking loss.

To understand the interrelationship between some specific starch characteristics (swelling power, solubility and amylose content) and water-holding capacities, the results about mathematical trends by fitting the linear equation were described in Figure 7A–F. It was evident that the linear equation could well fit the data between solubility and drying loss, with the high R^2^ value (0.956) (Figure 7C). Similarly, the better fitting curve (R^2^, 0.812) by linear equation was observed between amylose content of starch and cooking loss of sausage, as presented in Figure 7F. The remaining fitting curves had R^2^ values ranging from 0.653 to 0.756, indicating that the linear equation could reflect the underlying trend to a certain extent. These results meant that the physicochemical properties of starch have a direct impact on its water-holding function in meat products. Starch with high solubility (e.g., PS and SPS) may bind more tightly with water, thereby significantly reducing water loss during the drying process. Meanwhile, a higher amylose content may contribute to the formation of a more stable gel structure, which helps better retain moisture during cooking. These findings further confirmed that PS and SPS could develop into excellent at replacers for the meat food industry to meet specific texture and water-retention requirements.

In this study, a systematic evaluation approach was adopted to investigate the potential of five native starches as fat replacers in low-fat pork sausages. The findings revealed that PS and SPS exhibited superior physicochemical properties, including high amylose content, solubility, and swelling power. These properties significantly enhanced the water-retention capacity of the sausages, resulting in lower cooking loss and drying loss, maintaining good texture properties, and not adversely affecting the sensory attributes of the sausages. These results provide valuable insights into the development of healthier low-fat meat products without compromising on quality. To further advance the application of native starches in low-fat meat products and enhance their sustainability and cost-effectiveness, the future work will be conducted to (1) optimize the starch addition amount as well as the sausage-making and cooking processes; (2) combine more objective evaluation methods (such as gas chromatography–mass spectrometry or high-performance liquid chromatography) to analyze the flavor components and texture properties of sausages. Meanwhile, complement these with subjective evaluations conducted by rigorously screened and trained assessors to obtain more comprehensive and accurate research results; (3) design long-term storage experiments to evaluate the impact of starch as a fat substitute on quality indicators of sausages, such as color, texture, and flavor, as well as the long-term stability of starch; (4) conduct comprehensive life cycle assessments (LCAs) to evaluate the environmental impact of using native starches as fat replacers in low-fat meat products, from raw material production to processing, distribution, consumption, and disposal.

## 4. Conclusions

This study demonstrated the feasibility of using five native starches as functional fat replacers in low-fat pork sausages. Potato starch (PS) and sweet potato starch (SPS), despite their high amylose content (30.06% and 28.60%), demonstrated superior solubility (33.17% for PS) and swelling capacity (46.11 g/g for PS) due to their loose B/C-type crystalline structures, which enhanced water absorption and gel formation. These properties enabled PS and SPS to retain moisture effectively during thermal processing, reducing cooking loss (2.23% and 2.52%) and drying loss (6.75% and 7.03%) compared to controls. Additionally, PS and SPS improved sausage hardness and springiness, forming stable gel networks that maintained structural integrity without compromising toughness. Sensory analysis confirmed that PS/SPS did not alter taste or odor, except for rice starch (RS), which encapsulated flavors uniquely. These results confirmed that PS and SPS, as low-cost raw materials, can serve as effective fat substitutes, addressing health and quality demands in low-fat meat products. Overall, this research provided a scientific basis for the selection of starch-based fat replacers in the meat industry and offered a practical solution to develop healthier sausages that meet consumer demand for low-fat options.

## Figures and Tables

**Table 1:** Amylose content and chain-length distributions of amylose and amylopectin of five starches (*n* = 3, *p* < 0.05).

Sample	Amylose (%)	X ≤ 13 (%)	13 < X ≤ 24 (%)	24 < X ≤ 36 (%)	36 < X ≤ 100 (%)	100 < X ≤ 1000 (%)	1000 < X ≤ 5000 (%)	X > 5000 (%)
RS	21.93 ± 0.27 ^d^	19.14 ± 0.15 ^a^	27.89 ± 0.21 ^a^	12.23 ± 0.26 ^a^	18.81 ± 0.23 ^d^	8.63 ± 0.21 ^d^	8.34 ± 0.20 ^b^	4.96 ± 0.16 ^d^
SPS	30.06 ± 0.38 ^a^	10.73 ± 0.23 ^d^	21.18 ± 0.25 ^d^	12.24 ± 0.29 ^a^	25.80 ± 0.33 ^a^	13.50 ± 0.21 ^b^	8.52 ± 0.19 ^b^	8.04 ± 0.35 ^b^
CS	26.29 ± 0.19 ^bc^	16.51 ± 0.20 ^b^	26.44 ± 0.22 ^ab^	12.46 ± 0.16 ^a^	18.30 ± 0.19 ^d^	17.32 ± 0.29 ^a^	6.91 ± 0.13 ^c^	2.06 ± 0.19 ^e^
PS	28.60 ± 0.14 ^b^	14.02 ± 0.33 ^c^	24.80 ± 0.18 ^c^	12.50 ± 0.17 ^a^	20.10 ± 0.32 ^c^	11.42 ± 0.15 ^c^	10.32 ± 0.27 ^a^	6.86 ± 0.11 ^c^
TS	26.08 ± 0.22 ^c^	15.45 ± 0.29 ^b^	23.36 ± 0.26 ^c^	11.99 ± 0.22 ^b^	23.13 ± 0.16 ^b^	8.64 ± 0.22 ^d^	5.83 ± 0.19 ^d^	11.61 ± 0.08 ^a^

Note: values with different letters are significantly different. RS, PS, CS, SPS, TS represent rice starch, potato starch, corn starch, sweet potato starch, and tapioca starch, respectively.

**Table 2:** The pasting properties of five starches (*n* = 3, *p* < 0.05).

Sample	T_p_ (℃)	Peak Viscosity (mPa∙s)	Setback Viscosity (mPa∙s)	Breakdown Viscosity (mPa∙s)	Trough Viscosity (mPa∙s)	Final Viscosity (mPa∙s)
RS	90.55 ± 0.03 ^a^	667.32 ± 5.15 ^d^	676.28 ± 3.34 ^a^	227 ± 7.33 ^d^	460 ± 12.45 ^d^	1136 ± 5.53 ^c^
PS	76.00 ± 0.07 ^b^	912.11 ± 12.52 ^c^	478.56 ± 10.43 ^c^	354 ± 6.45 ^b^	558 ± 7.44 ^c^	1036 ± 13.34 ^d^
CS	92.65 ± 0.07 ^a^	694.64 ± 11.73 ^d^	289.27 ± 5.71 ^d^	199 ± 3.43 ^d^	495 ± 6.68 ^d^	784 ± 5.56 ^e^
SPS	75.30 ± 0.09 ^b^	1137.36 ± 17.86 ^b^	455.19 ± 12.85 ^c^	278 ± 18.45 ^c^	859 ± 3.44 ^b^	1314 ± 7.21 ^b^
TS	68.55 ± 0.06 ^c^	5349.19 ± 20.28 ^a^	543.45 ± 1.32 ^b^	3280 ± 23.57 ^a^	1529 ± 11.34 ^a^	2072 ± 12.65 ^a^

Note: values with different letters are significantly different. RS, PS, CS, SPS, TS represent rice starch, potato starch, corn starch, sweet potato starch, and tapioca starch, respectively.

**Table 3:** Sensitivity of PEN3 electronic nose sensors to various substances.

Array Number	Sensor	Substances for Sensing
S1	W1C	Aromatic compounds
S2	W5S	Nitrogen oxides
S3	W3C	Ammonia, Aromatic molecules
S4	W6S	Hydride
S5	W5C	Olefins, Aromatics, Polar molecules
S6	W1S	Taxanes
S7	W1W	Sulfur compound
S8	W2S	Detecting alcohols and some Aromatic compounds
S9	W2W	Aromatic compounds, Organic compounds of sulfur
S10	W3S	Alkanes and Aliphatics

**Table 4:** The effects of adding different types of starch on the color-difference values of sausages (*n* = 3, *p* < 0.05).

Color Difference	NC	LFC	RS	PS	CS	SPS	TS
L*	52.57 ± 0.41 ^e^	49.26 ± 0.41 ^f^	54.99 ± 1.7 ^d^	59.16 ± 2.05 ^a^	55.14 ± 1.69 ^d^	58.79 ± 1.86 ^b^	57.19 ± 1.97 ^c^
a*	5.98 ± 0.73 ^a^	3.53 ± 0.77 ^b^	4.45 ± 0.08 ^e^	4.91 ± 1.18 ^b^	4.67 ± 0.73 ^d^	4.93 ± 0.27 ^c^	4.11 ± 0.22 ^f^
b*	20.37 ± 1.34 ^b^	21.22 ± 1.21 ^a^	18.71 ± 1.14 ^c^	17.63 ± 2.75 ^d^	17.68 ± 2.23 ^d^	17.78 ± 2.67 ^d^	17.93 ± 1.53 ^d^

Note: L*, a* and b* represented luminance value, redness value and yellowness value, respectively; values with different letters are significantly different. NC, LFC, RS, PS, CS, SPS, TS represent normal control, low-fat control, rice starch, potato starch, corn starch, sweet potato starch, and tapioca starch treatments for pork sausages, respectively.

## Data Availability

The original contributions presented in this study are included in the article. Further inquiries can be directed to the corresponding authors.

## Appendix A

**Table A1:** The formulations of meat and starch emulsion.

Components	NC (g)	LFC (g)	Different Starch Groups (g)
Pork hind leg meat	60	69	60
Pork backfat	30	21	21
Ice water	10	10	10
Starch	0	0	9
Total mass	100	100	100

Note: NC and LFC represent normal control and low-fat control treatments for pork sausages, respectively.

**Table A2:** The crystallinity properties of the five starches.

Starch	RS (%)	PS (%)	CS (%)	SPS (%)	TS (%)
Crystallinity (%)	16.97 ± 0.21 ^c^	14.52 ± 0.14 ^e^	19.69 ± 0.11 ^b^	15.85 ± 0.16 ^d^	21.39 ± 0.23 ^a^

Note: values with different letters are significantly different (*p* < 0.05); RS, PS, CS, SPS, TS represent rice starch, potato starch, corn starch, sweet potato starch, and tapioca starch, respectively.
